# Reply to Fluegge, K.; Fluegge, K. Comment on “Frye et al. Air Pollution and Maximum Temperature Are Associated with Neurodevelopmental Regressive Events in Autism Spectrum Disorder. *J. Pers. Med.* 2022, *12*, 1809”

**DOI:** 10.3390/jpm15080382

**Published:** 2025-08-15

**Authors:** Richard E. Frye

**Affiliations:** Autism Discovery and Treatment Foundation, Phoenix, AZ 85050, USA; drfrye@autismdiscovery.org or info@autismdiscovery.org

I would like to thank Fluegge and Fluegge for their comments on our study which identified air pollution and maximum temperature as potential environmental factors associated with neurodevelopmental regression (NDR) in children with autism spectrum disorder (ASD) [1,2]. Our study aimed to help define this enigmatic phenomenon which is poorly understood. NDAR is a subtype of ASD in which an apparently typically developing child loses social and communication skills, either suddenly or over a period of weeks to months and develops the repetitive and restricted behavior associated with ASD. While fever and seizure are identified triggers in some cases, in most cases, a trigger is never identified [2].

Our recent studies have linked NDR to a unique state of mitochondrial function in which mitochondria are in a state of overdrive, with the electron transport chain functioning at approximately twice that of controls [3]. Our cell line model of this state of mitochondrial function demonstrates that this high respiratory state leads to vulnerability of the mitochondria such that a small physiological stress results in the mitochondria losing the ability to produce energy [3]. Interestingly, we have linked prenatal environmental factors such as air pollution as measured by PM_2.5_ and in utero concentrations of the nutritional metals zinc and manganese as factors which can induce this mitochondria state long term [3]. Given the significant effect of air pollution on the mitochondria during the prenatal period, it was hypothesized that this same factor could trigger NDR when the mitochondria was in this vulnerable state. Although our study supports this hypothesis, as Fluegge and Fluegge point out, the story is much more complex.

PM_2.5_ refers to particulate matter that is 2.5 µm or smaller in the atmosphere. Thus, this number is not specific to any one compound and represents a complex mixture of pollutants which can have different effects. For example, while black carbon absorbs solar radiation, resulting in increased temperature, sulfate and nitrate particles reflect sunlight, resulting in cooler temperatures. Furthermore, several environmental variables are closely interrelated and statistically coupled, so measuring one variable, such as PM_2.5_, may not provide a full account of the important environmental factors. There are well-known interactions between the major environmental factors of greenhouse gases, such as carbon dioxide and nitrous oxide (N_2_O), temperature, and air pollution (e.g., PM_2.5_). Greenhouse gases trap heat in the atmosphere, increasing temperatures which result in reduced precipitation and PM_2.5_ accumulation. Increased temperatures accelerate the production of ozone from nitrogen oxides and volatile organic compounds. This creates a vicious cycle which further increases temperature and secondarily increases air pollution.

The best environmental models would include multiple measurements of greenhouse gases and specific compounds in the atmosphere. However, for larger-scale studies that analyze environmental data across the United States, studies are limited by the measurements collected by the Environmental Protection Agency’s Air Quality System. This system has its limitations, as the measurements available depend on when the sensors were upgraded to measure more environmental variables. As such, our study was only able to include PM_2.5_, ozone, precipitation, and temperature [2]. Interestingly, the exposure variables were different depending on whether the ASD participant had an identified trigger to the NDR or not. For those without an identified trigger, air pollution was a risk factor for NDR, while maximum temperature was protective of experiencing NDR. For those with an identified trigger, air pollution did not appear to be a significant factor, but maximum temperature was a risk factor, while ozone and precipitation were protective variables.

Although we did not measure nitrous oxide (N_2_O) in our study, we found that lower ozone was a risk factor for NDR in individuals with a trigger. As previously mentioned, ozone is produced from the combination of nitrogen oxide (NxO) and volatile organic compounds, so lower ozone would be consistent with higher levels of nitrous oxide (N_2_O) which have not been converted to ozone in those with a trigger. Additionally, maximum temperature was found to be associated with a greater risk of NDR in those with a trigger. Interestingly, heat stimulates the release of nitrous oxide from soil microbes [4]. Since the most common triggers found for children with NDR are fever and seizure, this pattern is consistent with the reports of Fluegge and Fluegge, which link fever and seizure triggers of NDR to nitrous oxide.

A characteristic of ASD is high levels of reactive species such as reactive oxygen species (ROS) and reactive nitrogen species (RNS). RNS tend to be especially damaging to the cell. In individuals with ASD, high levels of RNS can be produced by physiological processes when tetrahydrobiopterin (BH_4_) is depleted. Specifically, a lack of BH_4_ uncouples nitrous oxide synthase, resulting in the production of peroxynitrite (NO_3_^−^), a highly reactive species, instead of nitric oxide (NO) [5]. Interestingly, BH_4_ depletion is associated with many neurodevelopmental disorders, including ASD [5]. Increased levels of NO_3_^−^ can lead to mitochondrial dysfunction, which is associated with NDR. Treatment with BH_4_ can favorably shift the redox pterin redox balance, increasing BH_4_ and restoring healthy NO synthesis [5].

Nitric oxide (NO) is a transient paracrine modulator of vascular tone and a neurotransmitter with important physiological roles in the body. However, nitric oxide (NO) can act as a reversible competitive inhibitor of mitochondrial function by occupying cytochrome c oxidase, the enzyme in the electron transport chain that uses oxygen as a substrate. This decreases the production of ATP and diverts oxygen to respiratory processes which increase ROS generation. NO also inhibits complex I of the electron transport chain, resulting in further mitochondrial dysfunction. Nitric oxide (NO) uses cytochrome c oxidase to produce nitrous oxide (N_2_O). Nitrous oxide (N_2_O), which is a common anesthetic, has been shown to inhibit mitochondrial function in the heart, liver, kidney, and brain as well as inactivate methionine synthetase and decrease formyltetrahydrofolates. Additionally, exposing the developing rat brain to anesthesia, including nitrous oxide, at the peak of synaptogenesis causes protracted injury to mitochondria, including an increase in complex IV activity, a pattern consistent with human prenatal exposure to air pollution in children with NDR.

Thus, exposure to several environmental factors can stress mitochondrial physiology and increase oxidative species, resulting in long-term mitochondrial changes and cellular damage. Nitrous species such as nitrous oxide (N_2_O) in the atmosphere may be one of the prominent environmental triggers of mitochondrial dysfunction. The idea that N_2_O may prevent NDR is antithetical to the medical and scientific evidence for individuals with mitochondrial disorders. As Fluegge and Fluegge discuss in their letter, N_2_O interferes with vitamin B12 metabolism. Also, because there is a high concentration of reactive species in those with mitochondrial disorders, nitrous species commonly become oxidized into peroxynitrite, which is highly reactive, easily damaging cellular structures. This is the reason that N_2_O is contraindicated as an anesthetic in those with mitochondrial disease. In fact, mitochondrial diseases characterized by a lack of nitric oxide (NO) are treated with arginine and/or citrulline to promote the local production of NO rather than supplementing with a nitrous species [6]. Further research is needed into the environmental components that adversely affect the developing brain, as well as methods to protect the developing child from these adversities.
